# Calculation method and predictive analysis of flexural capacity of reinforced concrete beams strengthened with carbon fiber-reinforced polymer sheets applied to the side surfaces

**DOI:** 10.1371/journal.pone.0353001

**Published:** 2026-07-02

**Authors:** Xiang Liu

**Affiliations:** School of Civil Engineering, Liaodong University, Dandong, Liaoning, China; Universiti Teknologi Malaysia, MALAYSIA

## Abstract

In response to the situation where it is not allowed to stick CFRP cloth at the bottom of a concrete beam and stick it on both sides of the beam, this article analyzes the factors that affect the ultimate flexural bearing capacity of reinforced concrete beams reinforced with CFRP on the side, and provides a calculation method for the flexural bearing capacity of reinforced concrete beams reinforced with CFRP on the side; At the same time, for the convenience of calculation, this paper explores the comprehensive consideration of the tensile force of carbon fiber cloth pasted on the side and the corresponding correction factor *η*_f_ of the force arm, and analyzes it by fitting a quadratic trend function with the ratio of CFRP pasting height to beam height (*h*_f_/*h*). Based on this, the calculation methods for the bending capacity of carbon fiber cloth pasted on the bottom surface according to the “Code” and the bending capacity of carbon fiber cloth pasted on the bottom surface according to the quadratic trend function are proposed. Research has shown that using CFRP to reinforce reinforced concrete beams on the side can effectively improve the flexural bearing capacity. After comparative analysis, the calculation results of three calculation methods are in good agreement with the experimental values; The correction coefficient *η*_f_ increases with the increase of the ratio of the bonding height to the beam height (*h*_f_/*h*). When the ratio of the bonding height to the beam height (*h*_f_/*h*) exceeds 0.25, the value of the correction coefficient *η*_f_ increases significantly; Especially when the ratio of the pasting height to the beam height (*h*_f_/*h*) exceeds 0.5, it is recommended to calculate the flexural bearing capacity of carbon fiber cloth pasted on the bottom surface according to the proposed quadratic trend function for *η*_f_; At the same time, it is recommended to consider the reduction of the cross-sectional area of carbon fiber cloth as compensation when determining the flexural bearing capacity of reinforced concrete beams with carbon fiber cloth pasted on the side according to the calculation of the beam bottom. In order to reduce errors, the utilization coefficient of *ψ*_f_ is no longer limited. Theoretical analysis shows that there are critical values for the bonding height and thickness of carbon fiber cloth used for reinforcement. When these exceed the critical value, the effect on enhancing load-bearing capacity becomes insignificant or even declines.

## 1. Introduction

**Fig 1:**
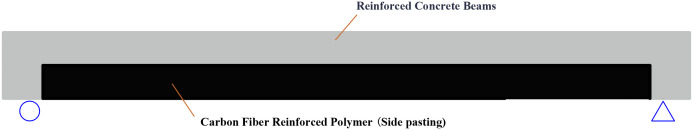
Reinforcement of a reinforced concrete beam with CFRP bonded to the side surface.

**Fig 2:**
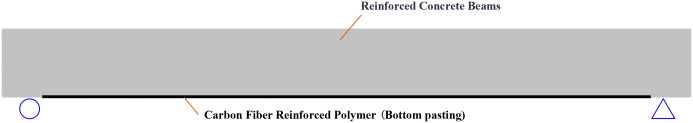
Reinforcement of a reinforced concrete beam with CFRP bonded to the bottom surface.

The large-scale construction model in the construction engineering sector has begun to exhibit a decelerating trend. Consequently, issues arising from structural aging and functional obsolescence of existing buildings are drawing increasing attention. Structural strengthening and repair carried out on the basis of existing structures offer an effective solution. Among various strengthening techniques, carbon fiber reinforced polymer reinforcement has emerged as a high-tech, widely adopted method for enhancing structural capacity. Specifically, bonding CFRP sheets onto concrete surfaces can significantly improve the flexural performance of reinforced concrete members. Currently, extensive research both domestically and internationally has been conducted on bottom-bonded CFRP strengthening of RC beams [1–19]. However, practical constraints including the presence of pipes, live electrical conduits adjacent to the beam soffit, or pre-constructed masonry walls and other obstructions beneath the beam often render bottom-bonding infeasible. In such cases, side-bonding of CFRP sheets becomes a viable alternative. Accordingly, systematic investigation into the flexural behavior particularly the ultimate flexural capacityof RC beams strengthened with side-bonded CFRP is both necessary and timely. Drawing upon existing literature: Tomoki Kawarai et al. [20]employed the method of bonding FRP sheets to the tensile side of the beam’s bottom to enhance the impact resistance of reinforced concrete beams under bending conditions. Subsequently, low-speed impact load tests (referred to as impact load tests) using a 300 kg steel weight were conducted on beams reinforced with CFRP sheets of varying area and mass to investigate the failure modes of the beams at their ultimate state.Cui Shiqi et al. [21] experimentally investigated the flexural capacity of concrete beams strengthened with side-bonded CFRP, analyzing its effects on bending strength, deformation, and ductility; based on test results, they derived a practical formula for calculating the flexural capacity of side-strengthened beams. Zhang Jiwen et al. [22,23] conducted experimental studies on the structural strengthening performance of side-bonded CFRP sheets applied to four under-reinforced RC beams. After inducing pre-cracks, CFRP sheets were bonded to the beam sides. Their study focused on the strengthened beams’ load-carrying capacity, ductility, deformation, crack development, and the influence of different CFRP bonding configurations; subsequently, they proposed a practical calculation method for the flexural capacity of side-strengthened beams. Wang Yuqing et al. [24] performed experimental and analytical studies on the flexural behavior of RC beams strengthened with side-bonded CFRP sheets (CFRPS), examining their effects on flexural capacity, stiffness, and crack patterns, and developed corresponding flexural capacity calculation models. Chen Xujun et al. [25] analyzed the flexural capacity of FRP-side-strengthened RC beams, discussed failure modes and design requirements based on experimental data, and—within the framework of the plane-section assumption—proposed a practical calculation method for the nominal flexural capacity of FRP-side-strengthened members. Despite these valuable contributions, several limitations persist across the aforementioned studies: (i) the reduction coefficient for the flexural capacity of RC beams strengthened with side-bonded CFRP is often provided only as an empirical reference value, lacking rigorous theoretical justification; (ii) the proposed flexural capacity calculation formulas are overly complex and cumbersome, impairing clarity and practical applicability; and (iii) insufficient attention has been paid to the physical interpretation and parametric sensitivity of the amplification factor *η*_f_, which accounts for the correction of the tensile resultant force and its moment arm of the side-bonded CFRP; notably, the constraint on the effective bonding height *h*_f_/*h* remains inadequately addressed. To address these gaps, this paper proposes simplified yet theoretically grounded calculation formulas for the flexural capacity of RC beams strengthened with CFRP sheets bonded at various side locations. For cases where side-bonded strengthening is equivalently converted to bottom-bonded strengthening, we provide a comprehensive analysis of the amplification factor *η*_f_, incorporating corrections for the CFRP tensile resultant and its lever arm, and validate our approach using relevant experimental data. Furthermore, key parameters influencing the ultimate flexural capacity of side-bonded CFRP-strengthened RC beams are identified and systematically examined. Schematic illustrations of side-bonded CFRP strengthening of RC beams and bottom-bonded CFRP strengthening of RC beams are presented in Fig 1 and 2, respectively.

## 2. Determination of the ultimate flexural capacity of reinforced concrete beams strengthened with side-bonded carbon fiber-reinforced polymer sheets

When reinforcing reinforced concrete beams with carbon fiber-reinforced polymer composites, in addition to satisfying the fundamental assumptions for sectional flexural capacity calculation stipulated in the current national standard *Code for Design of Concrete Structures* (GB 50010–2010) [26], the following provisions shall also be observed:

(1) The stress–strain relationship of the fiber-reinforced polymer material shall be modeled as linear;
(2) When secondary loading effects are considered, the lagged strain in the FRP material shall be determined based on the initial stress state of the member prior to strengthening;
(3) At the ultimate limit state of flexural capacity, debonding failure between the strengthening material and the concrete substrate shall not occur.

### 2.1. For RC beams strengthened with side-bonded CFRP sheets (see Figs 3 and 4), the flexural capacity of the strengthened section shall be calculated using the following equations


$$\begin{array}{c} M\leq f_{y}A_{s}\left(h_{\text{0}}-\frac{x}{\text{2}}\right)+\psi_{f}f_{f}A_{f,l}\left(h-\frac{h_{f}}{\text{2}}-\frac{x}{\text{2}}\right) \end{array}$$ (1)

$$\begin{array}{c} \alpha_{\text{1}}f_{c}bx=f_{y}A_{s}+\psi_{f}f_{f}A_{f,l} \end{array}$$ (2)

$$\begin{array}{c} \psi_{f}=\frac{\left[0.8\varepsilon_{cu}\left(h-\frac{\text{1}}{\text{2}}h_{f}\right)/x\right]-\varepsilon_{cu}-\varepsilon_{fo}}{\varepsilon_{f}} \end{array}$$ (3)

$$\begin{array}{c} M\leq f_{y}A_{s}\left(h_{\text{0}}-\frac{x}{\text{2}}\right)+\psi_{f}f_{f}\sum_{i=1}^{n}A_{f,li}\left(h-\frac{h_{fi}}{\text{2}}-\frac{x}{\text{2}}\right)+{\sigma^{\prime }}_{y}{A^{\prime }}_{s}\left(\frac{x}{\text{2}}-a^{\prime }\right) \end{array}$$ (4)

$$\begin{array}{c} \alpha_{\text{1}}f_{c}bx+{\sigma^{\prime }}_{y}{A^{\prime }}_{s}=f_{y}A_{s}+\psi_{f}f_{f}A_{f,l} \end{array}$$ (5)

$$\begin{array}{c} \psi_{f}=\frac{\left[0.8\varepsilon_{cu}\left(h-\frac{\text{1}}{\text{2}}h_{f}\right)/x\right]-\varepsilon_{cu}-\varepsilon_{fo}}{\varepsilon_{f}} \end{array}$$ (6)

Where in $h_{f}=\frac{\sum_{i=1}^{n}f_{f}A_{f,li}h_{fi}}{\sum_{i=1}^{n}f_{f}A_{f,li}}$

**Fig 3:**
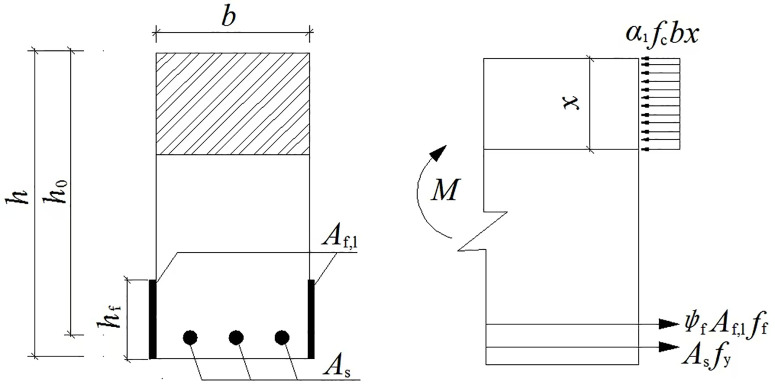
Calculation of the flexural capacity of reinforced concrete beams with CFRP bonded to the side surfaces.

**Fig 4:**
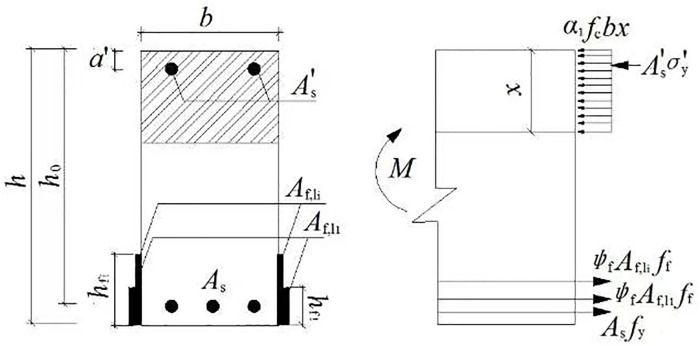
Flexural Capacity Calculation for Multi-Layer Side-Bonded CFRP-Strengthened RC Beams.

### 2.2. Determination of the Modified Amplification Factor *η*_f_

**Fig 5:**
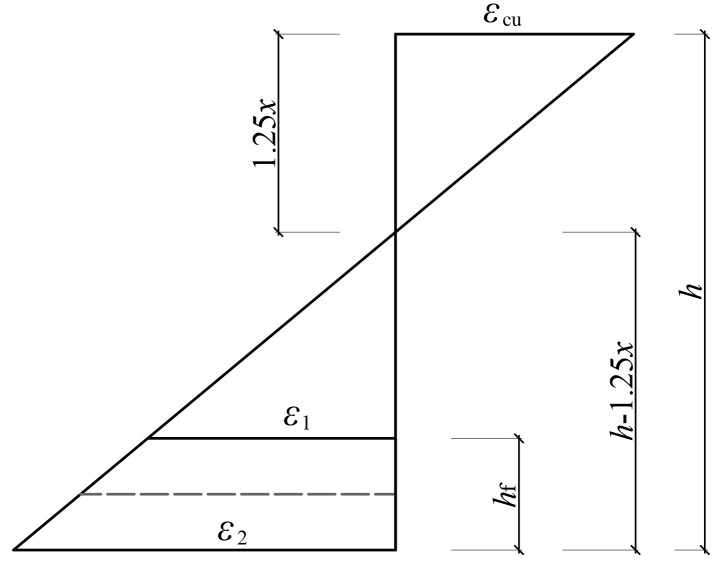
Strain distribution diagram based on the plane-sections-remain-plane assumption.


According to the assumption of a strain plane cross-section (see Fig 5), the ratio of the average strain at the upper and lower ends of the carbon fiber pasted on the side to the strain at the lower edge can be calculated [27], which is the correction factor *η*_f1_:

$$\begin{array}{c} ∵\frac{\varepsilon_{\text{1}}}{\varepsilon_{\text{2}}}=\frac{h-1.25x-h_{f}}{h-1.25x},x=\xi h_{\text{0}} \end{array}$$

$$\begin{array}{c} ∴\eta_{f1}=\frac{\frac{\varepsilon_{\text{1}}+\varepsilon_{\text{2}}}{\text{2}}}{\varepsilon_{\text{2}}}=\frac{\text{1}}{\text{2}}\cdot \left(\varepsilon_{\text{1}}+\varepsilon_{\text{2}}\right)=\frac{\text{1}}{\text{2}}\cdot \left(1+\frac{h-1.25x-h_{f}}{h-1.25x}\right)=1-\frac{0.5h_{f}}{h-1.25x}=1-\left(\frac{0.5}{1-1.25\xi h_{\text{0}}/h}\right)\left(\frac{h_{f}}{h}\right) \end{array}$$

Let $\beta_{\text{1}}=\frac{0.5}{1-1.25\xi h_{\text{0}}/h}$, Then $\eta_{f1}=1-\beta_{\text{1}}\left(\frac{h_{f}}{h}\right)$

Simultaneously, the ratio of the distance from the centroid of the resultant tensile force of the side-bonded CFRP sheet to the centroid of the compressive resultant force in the concrete, to the corresponding distance for a bottom-bonded CFRP sheet, shall be defined as the modification factor *η*_f2_.


$$\begin{array}{c} \eta_{f2}=\frac{\left(h-0.5x\right)-0.5h_{f}}{h-0.5x}=1-\left(\frac{0.5}{1-0.5\xi h_{\text{0}}/h}\right)\left(\frac{h_{f}}{h}\right) \end{array}$$

Let $\beta_{\text{2}}=\frac{0.5}{1-0.5\xi h_{\text{0}}/h}$, Then $\eta_{f2}=1-\beta_{\text{2}}\left(\frac{h_{f}}{h}\right)$

The overall modified amplification factor *η*_f_, which comprehensively accounts for both the tensile resultant force and its moment arm of the side-bonded CFRP sheet, is then obtained as follows:


$$\begin{array}{c} \eta_{f}=\frac{\text{1}}{\eta_{f1}\times \eta_{f2}}=\frac{\text{1}}{\left(1-\beta_{\text{1}}h_{f}/h\right)\times \left(1-\beta_{\text{2}}h_{f}/h\right)} \end{array}$$ (7)

Let $h_{\text{0}}=h/1.1,\xi =\xi_{b,f}.$

The relative depth of the compression zone at the balanced failure condition for the strengthened beam, denoted as *ξ*_b,f_, be taken as 0.85*ξ*_b_, where *ξ*_b_ is the corresponding value for the unstrengthened RC beam. Computed values of the parameters *β*_1_ and β2 for various grades of reinforcing steel are presented in Table 1; likewise, the corresponding values of the modification factor *η*_f_ are tabulated in Table 2.

**Table 1:** Computed values of the coefficients *β*₁ and *β*_2_.

Reinforcing Steel Grade	*ξ*_b_	*ξ*_b,f_	*β*_1_	*β*_2_
HPB300	0.576	0.4896	1.127	0.6431
HRB335, HRBF335	0.550	0.4675	1.067	0.6349
HRB400, HRBF400, RRB400	0.518	0.4403	1.001	0.6251
HRB500, HRBF500	0.482	0.4097	0.9356	0.6144

**Table 2:** Correction factor *η*ₚ for different grades of reinforcing steel.

*h*_f_/*h*Reinforcing Steel Grade	0.05	0.10	0.15	0.20	0.25	0.30	0.35	0.40	0.45	0.50
HPB300	1.095	1.204	1.332	1.482	1.659	1.872	2.131	2.451	2.855	3.377
HRB335, HRBF335	1.091	1.195	1.316	1.456	1.621	1.817	2.052	2.338	2.693	3.141
HRB400, HRBF400, RRB400	1.087	1.185	1.298	1.429	1.581	1.759	1.970	2.224	2.532	2.912
HRB500, HRBF500	1.082	1.175	1.281	1.403	1.542	1.704	1.894	2.119	2.387	2.712

“Code for Degin of Strengthening Concrete Structure”(GB50367−2013), provides only the coefficients β₁ and β₂ for members reinforced with HRB335 and HRB400 grade steel bars. Moreover, for conservatism, β₁ is fixed at 1.07 and β₂ at 0.63,without accounting for other steel grades. Consequently, in practical engineering applications involving steels of different grades (e.g., HRB500 or HPB300), designers must either adopt these conservative values originally intended for HRB335/HRB400 bars or derive new coefficients independently,a clear gap in the current code’s coverage. Table 1 comprehensively addresses this deficiency by listing the corresponding *β*₁ and *β*₂ values for all commonly used steel grades. Furthermore, the code’s amplification factor *η*_f_ is also derived solely from the *β*₁ and *β*₂ values associated with HRB335 and HRB400 steels and adopts the same conservative assumptions. It does not consider members reinforced with other steel grades (e.g., HRB500 or HPB300), resulting in a theoretical shortfall in engineering practice. Additionally, the code restricts the ratio of CFRP bonding height to beam depth (*h*_f_/*h*) to the narrow range of 0.05–0.25. To overcome these limitations, Table 2 not only incorporates *η*_f_ values calibrated for all steel grades but also extends the applicable *h*_f_/*h* range to 0.05–0.50. This extension aims to accommodate higher lateral bonding heights, yield more accurate *η*_f_ values, and mitigate the code’s inherent conservatism and scope limitations [28].

As indicated by the computational results, *η*_f_ increases monotonically with the ratio of the side-bonding height to the beam depth *h*_f_/*h*. Moreover, for a given *h*_f_/*h* ratio, *η*_f_ decreases gradually as the steel grade increases. Specifically, when *h*_f_/*h* increases from 0.05 to 0.25, *η*_f_ rises by approximately 50%; when *h*_f_/*h* further increases from 0.25 to 0.50, *η*_f_ increases by about 100%. An overestimated *η*_f_ leads to excessive reduction in the effective FRP area when converting from bottom-bonded to side-bonded configurations,thereby resulting in unnecessary material overdesign. Therefore, it is recommended that *h*_f_/*h* be limited to no more than 0.25 for side-bonded CFRP strengthening. This recommendation aligns well with the provision in the *Technical Code for Strengthening Concrete Structures*, which restricts side bonding to within one-quarter of the beam depth measured from the tension face.

**Fig 6:**
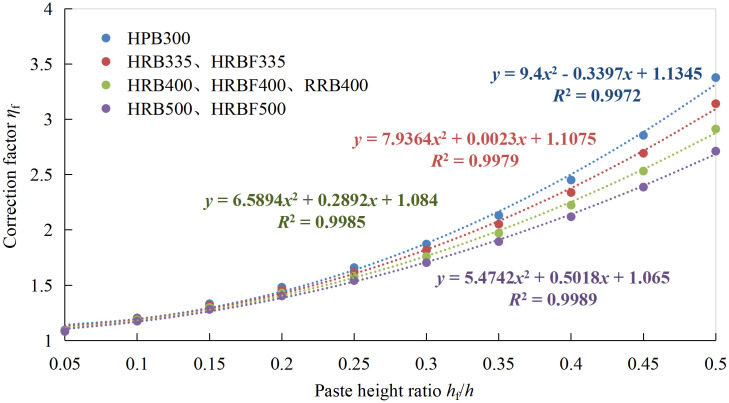
Fitted curve of the correction factor *η*_f_ versus the ratio of adhesive height to beam depth *h*_f_/h for various rebar grades.

As shown in Fig 6, for all steel grades considered, the functional relationship between *η*_f_ and *h*_f_/*h* exhibits a pronounced upward trend,particularly when *h*_f_/*h* exceeds 0.25. Furthermore, the coefficient of determination *R*^2^ for each fitted quadratic trend line is extremely close to unity, confirming high accuracy of the regression model.

### 2.3. For reinforced concrete beams strengthened by bonding carbon fiber–reinforced polymer sheets to the bottom surface[29] (Figs 7 and 8), the flexural capacity of the critical cross-section shall be determined according to the following equations


$$\begin{array}{c} M\leq f_{y}A_{s}\left(h_{\text{0}}-\frac{x}{\text{2}}\right)+\psi_{f}f_{f}A_{f,b}\left(h-\frac{x}{\text{2}}\right) \end{array}$$ (8)

$$\begin{array}{c} \alpha_{\text{1}}f_{c}bx=f_{y}A_{s}+\psi_{f}f_{f}A_{f,b} \end{array}$$ (9)

$$\begin{array}{c} \psi_{f}=\frac{\left[0.8\varepsilon_{cu}h/x\right]-\varepsilon_{cu}-\varepsilon_{fo}}{\varepsilon_{f}} \end{array}$$ (10)

Where in $A_{f,l}=\eta_{f}A_{f,b},A_{f,l}=2h_{f}t$

**Fig 7:**
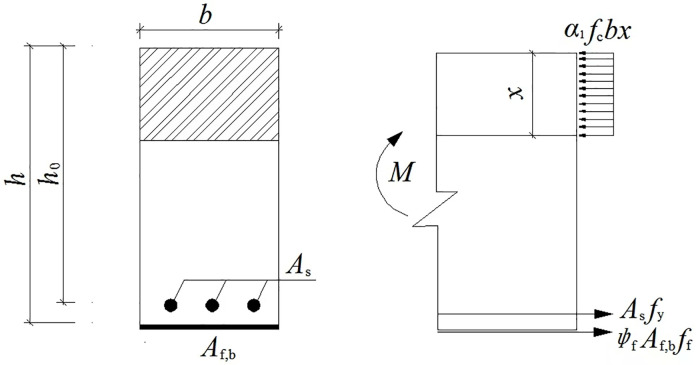
Flexural Capacity of an RC Beam Strengthened with Side-Bonded CFRP Laminates, Calculated Based on the Bottom Fiber Strain.

**Fig 8:**
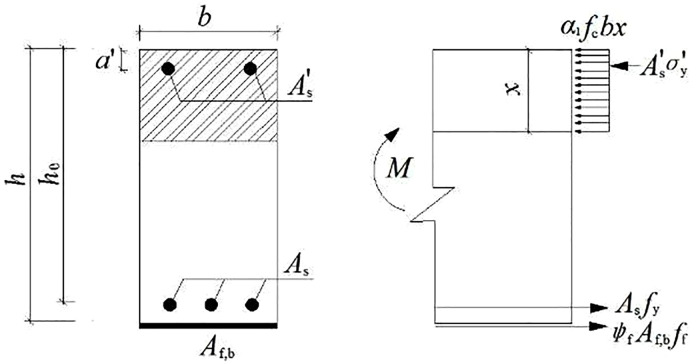
Flexural Capacity of an RC Beam Strengthened with Multi-Layer Side-Bonded CFRP Laminates, Calculated Based on the Bottom Fiber Strain.


$$\begin{array}{c} M\leq f_{y}A_{s}\left(h_{\text{0}}-\frac{x}{\text{2}}\right)+\psi_{f}f_{f}A_{f,b}\left(h-\frac{x}{\text{2}}\right)+{\sigma^{\prime }}_{y}{A^{\prime }}_{s}\left(\frac{x}{\text{2}}-a^{\prime }\right) \end{array}$$ (11)

$$\begin{array}{c} \alpha_{\text{1}}f_{c}bx+{\sigma^{\prime }}_{y}{A^{\prime }}_{s}=f_{y}A_{s}+\psi_{f}f_{f}A_{f,b} \end{array}$$ (12)

$$\begin{array}{c} \psi_{f}=\frac{\left[0.8\varepsilon_{cu}h/x\right]-\varepsilon_{cu}-\varepsilon_{fo}}{\varepsilon_{f}} \end{array}$$ (13)

Where in $\sum_{i=1}^{n}A_{f,li}=\sum_{i=1}^{n}\eta_{fi}\cdot A_{f,b},A_{f,l}=\sum_{i=1}^{n}A_{f,li}=2\sum_{i=1}^{n}h_{fi}t$

Notation: *M*—design bending moment of the strengthened member; *x*—depth of the concrete compression zone; if *x* < *2a,’* then *x* = *2a’*; *f*_*y*_—design yield strength of tension reinforcement; *f*_*c*_—design compressive strength of concrete in axial compression; *A*_s_—cross-sectional area of tension reinforcement; *A*_f,l_—the sum of the cross-sectional areas of each layer of fiber composite material that needs to be pasted on both sides of the beam; *A*_f,b_—the cross-sectional area of the fiber composite material determined by the calculation of the bottom surface of the beam but needs to be modified and pasted onto both sides of the beam; *b,* *h*—width and depth of the beam cross-section, respectively; *h*_f_—the equivalent vertical distance from the tension edge of the beam to the centroid of each layer of CFRP sheet bonded on the beam’s side surface;*ε*_cu_—ultimate compressive strain of concrete, taken as 0.0033;*ε*_f_—design tensile strain of CFRP;*ε*_f0_—lagged strain in the CFRP sheet induced by secondary loading effects; *ψ*_f_—strength utilization factor introduced to account for the fact that the actual tensile strain in side-bonded CFRP may fall short of its design value;*ψ*_f_ is capped at 1.0 when *ψ*_f_ > 1.0. In contrast, for bottom-bonded CFRP strengthening,*ψ*_f_ remains unchanged even when exceeding 1.0, reflecting the area reduction applied in the calculation; *t*—thickness of *t*he CFRP sheet.

The meanings of the symbols in the remaining formulas are shown in Table 3.

**Table 3:** Symbol Naming Table in Formulas.

*η*_f1_	The ratio of the average strain at the upper and lower ends of carbon fiber pasted on the side to the strain at the lower edge
*β*_1_	Consider the influence coefficient of the correction factor *η*_f1_ on the reinforcement of reinforced concrete beams with carbon fiber cloth pasted on the side
*η*_f2_	The ratio of the distance between the center of force of the carbon fiber cloth pasted on the side and the center of force of the concrete in the compression zone to the distance between the center of force of the carbon fiber cloth pasted on the bottom and the center of force of the concrete in the compression zone
*β*_2_	Consider the influence coefficient of the correction factor *η*_f2_ on the reinforcement of reinforced concrete beams with carbon fiber cloth pasted on the side
*η*_f_	Taking into account the tensile force of carbon fiber cloth pasted on the side and the corrected amplification factor of the corresponding force arm
*ξ*_b_	Height of relative boundary compression zone before reinforcement of reinforced concrete beams
*ξ*_b,f_	Relative limit compression zone height of reinforced concrete beams strengthened with carbon fiber cloth
*ψ*_f_	Side pasting considers the strength utilization coefficient introduced due to the actual tensile strain of carbon fiber cloth not reaching the design value
*A*_f,li_	The actual cross-sectional area of the i-th layer of fiber composite material that needs to be pasted on both sides of the beam
*A*_f,l_	The sum of the cross-sectional areas of each layer of fiber composite material that needs to be pasted on both sides of the beam
*h*_fi_	The vertical distance from the tension edge of the beam to the centroid of the i-th layer of carbon fiber reinforced polymer sheet bonded on the beam’s side surface
*h*_f_	The equivalent vertical distance from the tension edge of the beam to the centroid of each layer of CFRP sheet bonded on the beam’s side surface
*η*_fi_	Taking into account the tensile force of the i-th layer of carbon fiber cloth pasted on the side and the corrected amplification factor of the corresponding force arm
*A*_f,b_	The cross-sectional area of the fiber composite material determined by the calculation of the bottom surface of the beam but needs to be modified and pasted onto both sides of the beam

## 3. Factors Influencing the Ultimate Flexural Capacity of RC Beams Strengthened with Side-Bonded CFRP Sheets

Numerous factors influence the strengthening effectiveness of carbon fiber-reinforced polymer sheets. In addition to human-related factors such as construction practices [30], this paper primarily investigates and analyzes the relationships between the thickness and vertical bonding height of side-bonded CFRP sheets and the ultimate flexural capacity of reinforced concrete beams.The calculation model for the flexural bearing capacity of a reinforced concrete beam with carbon fiber cloth pasted on the side as shown in Figs 7 and 8, calculated based on the bottom surface, is obtained by substituting *A*_f,b_ = 2t*h*_f_/*η*_f_*A*_f,b_ = 2t*h*_f_/*η*_f_ into formula (9),


$$\begin{array}{c} x=\frac{f_{y}A_{s}+\psi_{f}f_{f}A_{f,b}}{\alpha_{\text{1}}f_{c}b}=\frac{f_{y}A_{s}}{\alpha_{\text{1}}f_{c}b}+\frac{\text{2}\psi_{f}f_{f}}{\alpha_{\text{1}}f_{c}b}\cdot \frac{h_{f}t}{\eta_{f}} \end{array}$$ (14)

Substituting Eq. (14) into Eq. (8) and rearranging yields a quadratic equation in terms of the CFRP sheet thickness *t* and the side-bonding height *h*_f_, relating the bending moment *M* to these two variables.


$$\begin{array}{c} M\leq -2\frac{\overset{\text{2}}{\underset{f}{\psi }}\overset{\text{2}}{\underset{f}{f}}}{\alpha_{\text{1}}f_{c}b}{\left(\frac{h_{f}t}{\eta_{f}}\right)}^{\text{2}}+\left(2\psi_{f}f_{f}h-2\frac{f_{y}A_{s}\psi_{f}f_{f}}{\alpha_{\text{1}}f_{c}b}\right)\left(\frac{h_{f}t}{\eta_{f}}\right)+0.909f_{y}A_{s}h-\frac{\text{1}}{\text{2}}\frac{\overset{\text{2}}{\underset{y}{f}}\overset{\text{2}}{\underset{s}{A}}}{\alpha_{\text{1}}f_{c}b} \end{array}$$

### 3.1. Relationship between the ultimate flexural capacity M and the side-bonding height hf

For CFRP-strengthened reinforced concrete beams with side-bonded CFRP sheets, when the side-bonding height *h*_f_ is held constant,

$M\leq a_{\text{1}}\overset{\text{2}}{\underset{f}{H}}+b_{\text{1}}H_{f}+c_{\text{1}}$, Let $\text{\textbackslash{}hspace\{0.17em}\}H_{f}=\frac{h_{f}}{h\eta_{f}}$

$H_{f}=\frac{h_{f}}{h\eta_{f}}=\frac{h_{f}/h}{\left(1-\beta_{\text{1}}h_{f}/h\right)\times \left(1-\beta_{\text{2}}h_{f}/h\right)},$ analysis indicates that *H*_f_ is a monotonically increasing function of *h*_f_*/h* within the physically reasonable range.

In the above equation:


$$\begin{array}{c} \begin{array}{c} a_{\text{1}}=-2\frac{\overset{\text{2}}{\underset{f}{\psi }}\overset{\text{2}}{\underset{f}{f}}h^{\text{2}}t^{\text{2}}}{\alpha_{\text{1}}f_{c}b}\mathrm{\backslash vspace}1mm\\ b_{\text{1}}=2\psi_{f}f_{f}h^{\text{2}}t-2\frac{f_{y}A_{s}\psi_{f}f_{f}ht}{\alpha_{\text{1}}f_{c}b}\mathrm{\backslash vspace}1mm\\ c_{\text{1}}=0.909f_{y}A_{s}h-\frac{\text{1}}{\text{2}}\frac{\overset{\text{2}}{\underset{y}{f}}\overset{\text{2}}{\underset{s}{A}}}{\alpha_{\text{1}}f_{c}b} \end{array} \end{array}$$

$$\begin{array}{c} \Delta =\overset{\text{2}}{\underset{\text{1}}{b}}-4a_{\text{1}}c_{\text{1}}=\overset{\text{2}}{\underset{f}{\psi }}\overset{\text{2}}{\underset{f}{f}}h^{\text{3}}t^{\text{2}}\left(4h-0.728\frac{f_{y}A_{s}}{\alpha_{\text{1}}f_{c}b}\right)>0 \end{array}$$

$$\begin{array}{c} \frac{c_{\text{1}}}{a_{\text{1}}}=\frac{\frac{\text{1}}{\text{2}}\frac{\overset{\text{2}}{\underset{y}{f}}\overset{\text{2}}{\underset{s}{A}}}{\alpha_{\text{1}}f_{c}b}-0.909f_{y}A_{s}h}{2\frac{\overset{\text{2}}{\underset{f}{\psi }}\overset{\text{2}}{\underset{f}{f}}h^{\text{2}}t^{\text{2}}}{\alpha_{\text{1}}f_{c}b}}=\frac{f_{y}A_{s}}{\overset{\text{2}}{\underset{f}{\psi }}\overset{\text{2}}{\underset{f}{f}}h^{\text{2}}t^{\text{2}}}\left(\frac{\text{1}}{\text{4}}f_{y}A_{s}-0.4545\alpha_{\text{1}}f_{c}bh\right)<0 \end{array}$$

**Fig 9:**
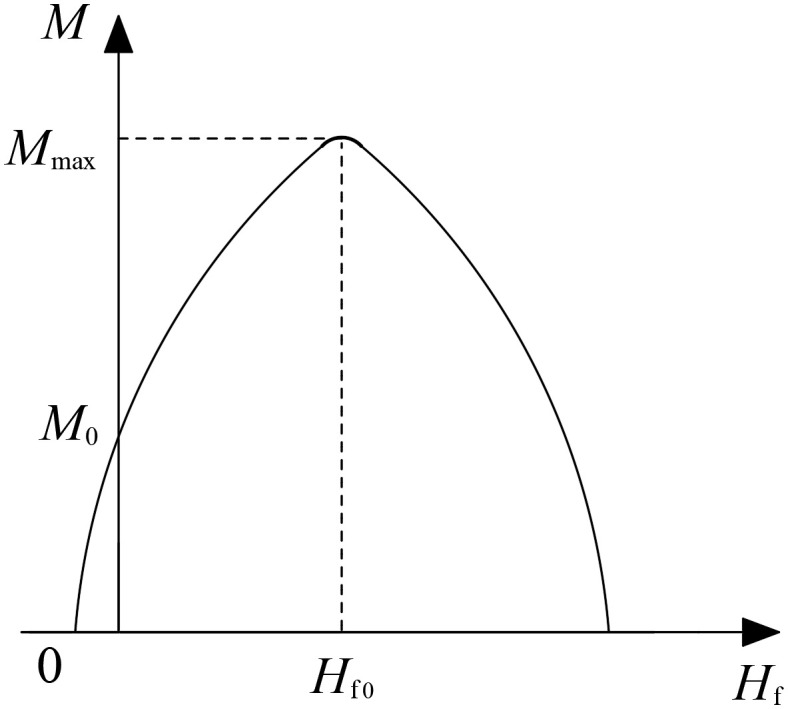
Relationship between the Ultimate Bending Moment *M* and the Height of the CFRP Laminate *H*_f._

As revealed by analytical examination, the equation possesses two real roots one positive and one negative. The negative root is physically meaningless and thus discarded; only the positive root is retained. As shown in Fig 9,

1. When 0 < *H*_f_ < *H*_f0_, the flexural capacity *M* of the strengthened beam increases with increasing *H*_f_;
2. When *H*_f_ > *H*_f0_, reinforced concrete beams strengthened with carbon fiber reinforced polymer sheets exhibit a counterintuitive reduction in flexural capacity *M* when the side-bonding height *h*_f_ exceeds a critical threshold *h*_f0_, i.e., as the ratio *h*_f_ increases beyond this point;
3. When *H*_f_ = *H*_f0_, the flexural capacity *M* attains its maximum value *M*_max_, at which point.

$$\begin{array}{c} H_{f0}=-\frac{b_{\text{1}}}{2a_{\text{1}}}=\frac{2\psi_{f}f_{f}h^{\text{2}}t-2\frac{f_{y}A_{s}\psi_{f}f_{f}ht}{\alpha_{\text{1}}f_{c}b}}{2\times 2\frac{\overset{\text{2}}{\underset{f}{\psi }}\overset{\text{2}}{\underset{f}{f}}h^{\text{2}}t^{\text{2}}}{\alpha_{\text{1}}f_{c}b}}=\frac{\text{1}}{\text{2}}\left(\frac{\alpha_{\text{1}}f_{c}bh-f_{y}A_{s}}{\psi_{f}f_{f}ht}\right) \end{array}$$

$$\begin{array}{c} M_{max}=\frac{4a_{\text{1}}c_{\text{1}}-\overset{\text{2}}{\underset{\text{1}}{b}}}{4a_{\text{1}}}=c_{\text{1}}-\frac{\overset{\text{2}}{\underset{\text{1}}{b}}}{4a_{\text{1}}} \end{array}$$

### 3.2. Relationship between the ultimate flexural capacity M and the CFRP sheet thickness t

For CFRP-strengthened reinforced concrete beams with side-bonded CFRP sheets, when the CFRP sheet thickness t is held constant,


$$\begin{array}{c} M\leq a_{\text{2}}t^{\text{2}}+b_{\text{2}}t+c_{\text{2}} \end{array}$$

In the above equation:


$$\begin{array}{c} \begin{array}{c} a_{\text{2}}=-2\frac{\overset{\text{2}}{\underset{f}{\psi }}\overset{\text{2}}{\underset{f}{f}}}{\alpha_{\text{1}}f_{c}b}{\left(\frac{h_{f}}{\eta_{f}}\right)}^{\text{2}}\\ b_{\text{2}}=\left(2\psi_{f}f_{f}h-2\frac{f_{y}A_{s}\psi_{f}f_{f}}{\alpha_{\text{1}}f_{c}b}\right)\left(\frac{h_{f}}{\eta_{f}}\right)\\ c_{\text{2}}=0.909f_{y}A_{s}h-\frac{\text{1}}{\text{2}}\frac{\overset{\text{2}}{\underset{y}{f}}\overset{\text{2}}{\underset{s}{A}}}{\alpha_{\text{1}}f_{c}b} \end{array} \end{array}$$

$$\begin{array}{c} △=\overset{\text{2}}{\underset{\text{2}}{b}}-4a_{\text{2}}c_{\text{2}}=\frac{\overset{\text{2}}{\underset{f}{\psi }}\overset{\text{2}}{\underset{f}{f}}\overset{\text{2}}{\underset{f}{h}}h^{\text{2}}}{\overset{\text{2}}{\underset{f}{\eta }}}\left(4-0.728\frac{f_{y}A_{s}}{\alpha_{\text{1}}f_{c}bh}\right)>0 \end{array}$$

$$\begin{array}{c} \frac{c_{\text{2}}}{a_{\text{2}}}=\frac{\frac{\text{1}}{\text{2}}\frac{\overset{\text{2}}{\underset{y}{f}}\overset{\text{2}}{\underset{s}{A}}}{\alpha_{\text{1}}f_{c}b}-0.909f_{y}A_{s}h}{2\frac{\overset{\text{2}}{\underset{f}{\psi }}\overset{\text{2}}{\underset{f}{f}}}{\alpha_{\text{1}}f_{c}b}{\left(\frac{h_{f}}{\eta_{f}}\right)}^{\text{2}}}=\frac{f_{y}A_{s}\overset{\text{2}}{\underset{f}{\eta }}}{\overset{\text{2}}{\underset{f}{\psi }}\overset{\text{2}}{\underset{f}{f}}\overset{\text{2}}{\underset{f}{h}}}\left(\frac{\text{1}}{\text{4}}f_{y}A_{s}-0.4545\alpha_{\text{1}}f_{c}bh\right)<0 \end{array}$$

**Fig 10:**
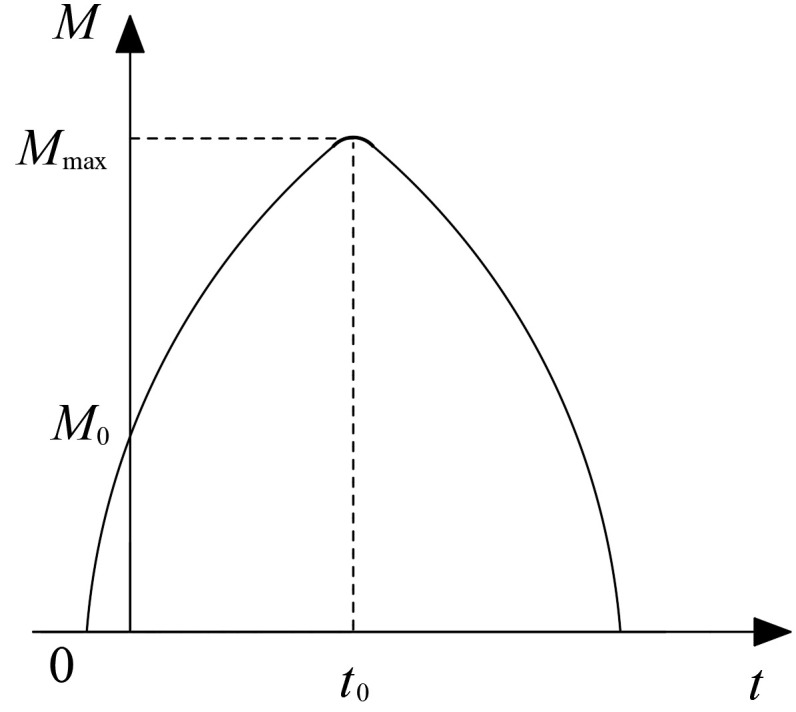
Relationship between the Ultimate Bending Moment *M* and the Thickness of the Bonded CFRP Laminate *t.*

As revealed by analytical examination, the equation possesses two real roots one positive and one negative. The negative root is physically meaningless and thus discarded; only the positive root is retained.As shown in Fig 10,

1. When 0 < *t* < *t*_0_, the flexural capacity M of the strengthened beam increases with increasing *t*;
2. When *t* > *t*_0_, further increasing the CFRP thickness t results in behavior analogous to over-reinforced failure in conventional reinforced concrete beams: the compressive concrete crushes prematurely, while the tensile steel yields and the CFRP remains below its allowable stress level consequently, the beam’s load-carrying capacity declines significantly;
3. When *t* = *t*_0_, the flexural capacity *M* reaches its maximum value *M*_max_, at which point

$$\begin{array}{c} t_{\text{0}}=-\frac{b_{\text{2}}}{2a_{\text{2}}}=\frac{\left(2\psi_{f}f_{f}h-2\frac{f_{y}A_{s}\psi_{f}f_{f}}{\alpha_{\text{1}}f_{c}b}\right)\left(\frac{h_{f}}{\eta_{f}}\right)}{2\times 2\times \frac{\overset{\text{2}}{\underset{f}{\psi }}\overset{\text{2}}{\underset{f}{f}}}{\alpha_{\text{1}}f_{c}b}{\left(\frac{h_{f}}{\eta_{f}}\right)}^{\text{2}}}=\frac{\text{1}}{\text{2}}\left(\frac{\alpha_{\text{1}}f_{c}bh-f_{y}A_{s}\eta_{f}}{\psi_{f}f_{f}h_{f}}\right) \end{array}$$

$$\begin{array}{c} M_{max}=\frac{4a_{\text{2}}c_{\text{2}}-\overset{\text{2}}{\underset{\text{2}}{b}}}{4a_{\text{2}}}=c_{\text{2}}-\frac{\overset{\text{2}}{\underset{\text{2}}{b}}}{4a_{\text{2}}} \end{array}$$

Collectively, the beam’s cross-sectional dimensions, longitudinal reinforcement configuration, concrete strength, and the mechanical properties of the CFRP material all influence both the required amount of CFRP and the maximum achievable flexural capacity.

## 4. Comparative analysis of calculated and experimental flexural capacities of RC beams strengthened with laterally bonded CFRP sheets

The flexural capacity calculation formula for CFRP-strengthened RC beams specified in the”Technical Code for Strengthening Concrete Structures”(GB 50367–2013) is theoretically grounded in the assumption of ideal ductile (i.e., tension-controlled) failure. It presumes sufficiently robust interfacial bond strength and allocates all safety margins exclusively to material strength capacities, while relying on strict geometric and mechanical constraints to preclude brittle debonding failure. Although the present study’s proposed calculation method for laterally bonded CFRP-strengthened RC beams retains the fundamental assumptions of the code’s sectional capacity model, it explicitly accounts for the following five distinct failure modes:① Sequential rupture of the CFRP sheet at the beam’s lateral bottom followed by crushing of the concrete in compression;② Yielding of the tensile reinforcement followed by concrete crushing in compression, while the CFRP tensile strain remains below its allowable limit;③ Yielding of the tensile reinforcement accompanied by CFRP tensile strain reaching its allowable limit, prior to concrete crushing in compression;④ Rupture of the lower portion of the CFRP sheet and debonding of its upper portion after steel yielding; ⑤ Simultaneous partial CFRP rupture and partial debonding following steel yielding and prior to complete concrete crushing in compression. However, the proposed method is not applicable to cases involving concrete cover separation and subsequent shear-induced CFRP debonding.

### 4.1. Applicability scope of the proposed flexural capacity calculation method for laterally bonded CFRP-strengthened RC beams

Due to the limited availability of published experimental data on side-bonded CFRP strengthening of reinforced concrete beams, this study compiled test data from domestic researchers including Cui Shiqi et al., Wang Yuqing et al., and Zhang Jiwen et al,as shown in Table 4. The flexural capacities predicted by the proposed analytical model for side-bonded CFRP strengthening were compared against these experimental results. As shown in Tables 5 and 6, the ratio of calculated to experimental values exhibits the following statistical characteristics: For the proposed side-bonding model: mean ratio *μ*_1_ = 0.99425, standard deviation *σ*_1_ = 0.0586, and coefficient of variation *C*_V1_ = 0.0589; For the conventional bottom-bonding model using the amplification factor *η*_f_ prescribed in the relevant design code: mean ratio *μ*_2_ = 0.94792, standard deviation *σ*_2_ = 0.0912, and coefficient of variation *C*_V2_=0.0962; For the bottom-bonding model using the proposed quadratic trend function to determine *η*_f_: mean ratio *μ*_3_ = 0.97633, standard deviation *σ*_3_=0.0514, and coefficient of variation *C*_V3_ = 0.0526. All three methods yield prediction ranges that are physically reasonable and exhibit high reliability. Notably, *μ*_1_ > *μ*_3_ > *μ*_2_ and *C*_V3_ < *C*_V1_ < *C*_V2_, indicating that the bottom-bonding model incorporating the proposed quadratic trend function for *η*_f_ delivers the highest accuracy and consistency, whereas the code-prescribed *η*_f_ approach yields the lowest performance.

**Table 4:** Parameter values influencing the flexural capacity of CFRP-strengthened reinforced concrete beams.

References	Specimen	Beam Width /mm	Beam Height /mm	Shear Span _/mm_	*f*_c_ /MPa	Steel Reinforcement Strength		Steel Reinforcement Area		CFRP Sheet			
						*F*_y_ /MPa	*f'*_y_ /MPa	*A*_s_ /mm^2^	*A'*_s_ /mm^2^	*f*_f_/MPa	n /layers	*h*_f_/mm	*t*/mm
Cui Shiqi et al.	S-Beam	150	250	750	20.1	310	―	339	―	3000	3	50	0.111
Wang Yuqing et al.	B11	100	200	500	19.97	374.41	424.03	101	57	3000	1	50	0.111
	B12	100	200	500	19.97	374.41	424.03	101	57	3000	1	50	0.111
	B21	100	200	500	19.97	374.41	424.03	101	57	3000	1	100	0.111
	B22	100	200	500	19.97	374.41	424.03	101	57	3000	1	100	0.111
	B32	100	200	500	19.97	374.41	424.03	101	57	2350	2	50	0.111
	B41	100	200	500	19.97	374.41	424.03	101	57	3000	1	150	0.111
	B42	100	200	500	19.97	374.41	424.03	101	57	3000	1	150	0.111
Zhang Jiwen et al.	BCF-1	150	350	700	15.5	350	350	66	66	3700	1	175	0.111
	BCF-2	150	350	700	15.5	350	350	66	66	3700	1 (1)	175 (90)	0.111
	BCF-3	150	350	700	15.5	350	350	66	66	3700	2	175	0.111
	BCF-4	150	350	700	15.5	350	350	66	66	3700	1 (3)	175 (90)	0.111

Notation “1(1)” denotes one layer bonded at a side-bonding height of 175 mm and one layer bonded at a side-bonding height of 90 mm; “1(3)” denotes one layer bonded at 175 mm and three layers bonded at 90 mm. In specimen B32, the CFRP effective stress *f*_f_ is taken as *f*_f_ = *E*_f_ × *ε* = 2.35 × 10^5^ × 0.01 = 2350MPa, because the test report explicitly states that failure was governed by concrete crushing in compression and steel yielding in tension, while the CFRP remained below its allowable strain limit.

**Table 5:** Comparison between experimental ultimate load values and calculated ultimate load values obtained using the proposed formulas for side-bonded and bottom-bonded CFRP strengthening.

References	Specimen	*P*_uE_ /kN	CFRP Side-Bonded Reinforced Concrete Beams					Reinforced Concrete Beams with CFRP Sheets Bonded to the Soffit				
			*h*_f_/*h*	*A*_f,l_ /mm^2^	*ψ*_f_	*P*_uC_ /kN	*P*_uC_/*P*_uE_	*η*_f_	*A*_f,b_ /mm^2^	*ψ*_f_	*P*_uC_ /kN	*P*_uC_/*P*_uE_
Cui Shiqi et al.	S-Beam	90.0	0.20	33.3	0.668	85.5	0.950	1.482	22.5	0.879	87.8	0.976
Wang Yuqing et al.	B11	42.5	0.25	11.1	0.939	44.4	1.045	1.659	6.7	1.121	41.1	0.967
	B12	45.0	0.25	11.1	0.939	44.4	0.987	1.659	6.7	1.121	41.1	0.913
	B21	48.0	0.50	22.2	0.758	51.4	1.071	3.377	6.6	1.741	50.9	1.060
	B22	53.0	0.50	22.2	0.758	51.4	0.970	3.377	6.6	1.741	50.9	0.960
	B32	52.0	0.25	22.2	0.939	55.5	1.067	1.659	13.4	1.121	50.4	0.969
	B41	51.0	0.75	33.3	0.569	49.0	0.961	12.48	2.7	2.524	40.8	0.800
	B42	54.3	0.75	33.3	0.569	49.0	0.902	12.48	2.7	2.524	40.8	0.751
Zhang Jiwen et al.	BCF-1	102.5	0.50	38.85	0.908	104.9	1.023	3.377	11.5	2.089	99.7	0.973
	BCF-2	122.5	0.50 (0.26)	38.85 (19.98)	0.734	129.6	1.058	3.377 (1.698)	23.3	1.423	130.4	1.064
	BCF-3	132.5	0.50	77.7	0.603	130.5	0.985	3.377	23.0	1.433	129.8	0.980
	BCF-4	175.0	0.50 (0.26)	38.85 (59.94)	0.550	159.6	0.912	3.377 (1.698)	46.8	0.961	168.3	0.962

Notation “0.50(0.26)” denotes a side-bonding ratio of 0.5 at 175 mm and 0.26 at 90 mm; “38.85(19.98)” denotes CFRP cross-sectional areas of 38.85 mm^2^ at 175 mm and 19.98 mm^2^ at 90 mm; “3.377(1.698)” denotes amplification factors of 3.377 at 175 mm and 1.698 at 90 mm.

**Table 6:** Comparison of the CFRP bottom-bonding flexural capacity formula incorporating the correction factor *η*_f_ calculated either via the proposed quadratic trend function or in accordance with the Code provisions.

References	Specimen	*h*_f_/*h*	Reinforced Concrete Beams with CFRP Sheets Bonded to the Soffit *η*_f_ computed using the proposed quadratic trend function					Reinforced Concrete Beams with CFRP Sheets Bonded to the Soffit *η*_f_ computed per GB 50367–2013				
			*η*_f_	*A*_f,b_ /mm^2^	*ψ*_f_	*P*_uC_ /kN	*P*_uC_/*P*_uE_	*η*_f_	*A*_f,b_ /mm^2^	*ψ*_f_	*P*_uC_/kN	*P*_uC_/*P*_uE_
Cui Shiqi et al.	S-Beam	0.20	1.443	23.1	0.872	88.4	0.982	1.482	22.5	0.879	87.8	0.976
Wang Yuqing et al.	B11	0.25	1.637	6.8	1.121	41.4	0.974	1.659	6.7	1.121	41.1	0.967
	B12	0.25	1.637	6.8	1.121	41.4	0.920	1.659	6.7	1.121	41.1	0.913
	B21	0.50	3.315	6.7	1.733	51.1	1.065	3.377	6.6	1.741	50.9	1.060
	B22	0.50	3.315	6.7	1.733	51.1	0.964	3.377	6.6	1.741	50.9	0.960
	B32	0.25	1.637	13.6	1.121	50.8	0.977	1.659	13.4	1.121	50.4	0.969
	B41	0.75	6.167	5.4	1.898	48.2	0.945	12.48	2.7	2.524	40.8	0.800
	B42	0.75	6.167	5.4	1.898	48.2	0.888	12.48	2.7	2.524	40.8	0.751
Zhang Jiwen et al.	BCF-1	0.50	3.315	11.7	2.070	100.4	0.980	3.377	11.5	2.089	99.7	0.973
	BCF-2	0.50 (0.26)	3.315 (1.682)	23.6	1.413	131.1	1.070	3.377 (1.698)	23.3	1.423	130.4	1.064
	BCF-3	0.50	3.315	23.4	1.420	130.7	0.986	3.377	23.0	1.433	129.8	0.980
	BCF-4	0.50 (0.26)	3.315 (1.682)	47.4	0.953	168.8	0.965	3.377 (1.698)	46.8	0.961	168.3	0.962

Comparative evaluation (Section 3) between calculated and experimental results reveals that when the bonding height-to-depth ratio *h*_f_/*h* exceeds 0.50 and reaches 0.75, the conventional approach i.e., determining *η*_f_ per the code’s prescribed values and applying it to a bottom-surface-based flexural capacity model,yields notably large errors. In contrast, both the modified bottom-surface-based method (employing *η*_f_ derived from the proposed quadratic trend function) and the newly developed lateral-bonding-specific calculation method produce significantly smaller errors. This indicates that the code-prescribed *η*f based bottom surface method has inherent limitations and is strictly valid only for *h*_f_/*h* ≤ 0.50. When *h*_f_/*h* > 0.50, it is recommended to adopt either (i) the modified bottom-surface method using *η*_f_ from the quadratic trend function, or (ii) the dedicated lateral-bonding calculation method proposed herein.

**Fig 11:**
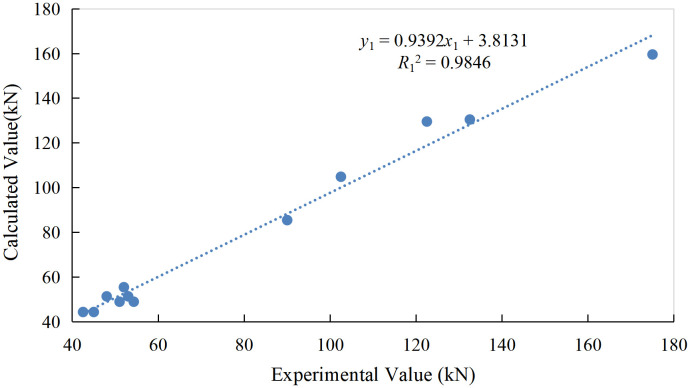
Linear regression plot of calculated versus experimental flexural capacities for beams strengthened with side-bonded CFRP sheets.

**Fig 12:**
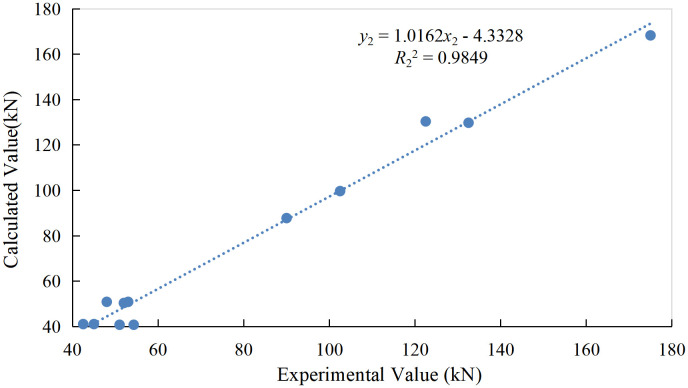
Linear regression plot of calculated versus experimental flexural capacities for beams strengthened with bottom-bonded CFRP sheets, using the correction factor ηf determined per the Code provisions.

**Fig 13:**
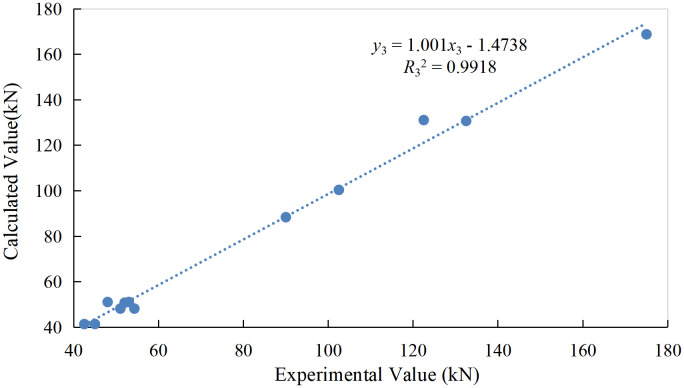
Linear regression plot of calculated versus experimental flexural capacities for beams strengthened with bottom-bonded CFRP sheets, using the correction factor *η*_f_ derived from the proposed quadratic trend function.

(1) As shown in Table 4, side-bonded CFRP strengthening effectively enhances the flexural capacity of reinforced concrete beams. However, under identical parameter conditions, a larger side-bonding ratio *h*_f_/*h* corresponds to a lower utilization factor *ψ*_f_ of the CFRP. Moreover, even when *h*_f_/*h* remains constant, *ψ*_f_ decreases with increasing number of CFRP layers.
(2) When calculating the flexural capacity of CFRP side-bonded reinforced concrete beams using the conventional method developed for soffit-bonded configurations, a pronounced increase in the amplification factor *η*_f_ is observed once the side-bonding ratio *h*_f_/*h* exceeds 0.50. Notably, at *h*_f_/*h*, *η*_f_ reaches 12.48, leading to significant underestimation of the effective CFRP area *A*_f,b_ and consequently large discrepancies between calculated ultimate flexural capacities and experimental results. Therefore, it is recommended that the side-bonding ratio *h*_f_/*h* be limited to 0.50 or less. In contrast, the *Code for Design of Strengthening Concrete Structures* (GB50367-2013) prescribes a more conservative upper limit of 0.25 for *h*_f_/*h* when applying the soffit-bonding calculation method，this restriction appears overly cautious. For cases where *h*_f_/*h* > 0.50, the amplification factor *η*_f_ is recommended to be determined via the proposed quadratic trend function. For instance, at *h*_f_/*h* = 0.75, *η*_f_ = 6.167 yields a calculated ultimate load *P*_uC_ = 48.2kN, which closely matches the experimental value and exhibits markedly reduced error. As shown in Table 5, adopting the quadratic trend function for *η*_f_ leads to smaller deviations between predicted and experimental flexural capacities compared to using the code-specified *η*_f_ values,particularly for *h*_f_/*h* > 0.50. Hence, the quadratic trend function is recommended for engineering applications.
(3) This study proposes a modification to the utilization factor *ψ*_f_ used in flexural capacity calculations for CFRP side-bonded beams: rather than capping *ψ*_f_ at 1.0 when *ψ*_f_ > 1.0, (as conventionally practiced), *ψ*_f_ is permitted to exceed unity to compensate for the effective reduction in CFRP cross-sectional area arising from non-uniform strain distribution. Imposing an artificial upper bound of *ψ*_f_ = 1.0 may otherwise introduce substantial errors relative to experimental data. This relaxation represents a novel contribution of the present work.
(4) As illustrated in Figs 11, 12, and 13, linear regression analyses between calculated and experimental flexural capacities yield determination coefficients of *R*_1_^2^ = 0.9846 (for side-bonded configurations), *R*_2_^2^ = 0.9849 (for soffit-bonded configurations using the code-specified *η*_f_, and *R*_3_^2^ = 0.9918 (for soffit-bonded configurations using the proposed quadratic *η*_f_. All three values are close to unity, indicating excellent model fit; among them, *R*_3_^2^ is the highest, confirming superior predictive accuracy of the quadratic *η*_f_ approach.

The ratios of calculated-to-experimental flexural capacities obtained using the three proposed CFRP strengthening formulas are consistently close to unity, demonstrating high computational precision and practical applicability to real-world structural strengthening projects.

### 4.2. Limitations and inapplicable cases of the proposed flexural capacity calculation method

Debonding failure of CFRP-strengthened RC beams is a sudden, brittle mode of failure. The proposed calculation method for laterally bonded CFRP-strengthened RC beams is not applicable to cases involving concrete cover separation and shear-induced CFRP debonding. In actual engineering design, due to the high variability and lack of formal codification of such failures, the code advocates a “prevention-only, no-calculation” strategy. Recognizing this, the present study acknowledges that, in cases where concrete cover separation and shear debonding occur, the actual ultimate load-carrying capacity is typically reduced. Therefore, for design purposes, the strengthened structural capacity should be conservatively assessed at 70% ~ 80% of the nominal calculated value. For instance, in the experimental dataset reported by Wang Yuqing et al., specimen B31 failed via CFRP debonding; its experimentally measured ultimate load was 38.0 kN. Using the average of the three calculation methods proposed herein,applied to specimen B32 (a comparable control specimen),the computed ultimate load was 52.2 kN. Applying the 70% ~ 80% reduction yields an estimated capacity range of 36.5 ~ 41.8 kN for B31, which closely aligns with its measured value of 38.0 kN. This agreement supports the reasonableness of the capacity reduction approach for debonding-dominated failures. Nevertheless, rigorous theoretical analysis and further experimental validation remain topics for future research.

## 5. Conclusions

(1) This study develops a modified amplification factor *η*_f_ that comprehensively accounts for both the tensile resultant force and its corresponding moment arm of side-bonded CFRP sheets. A quadratic trend function is fitted between *η*_f_ and the side-bonding ratio *h*_f_/*h*. Results indicate that *η*_f_ increases significantly as *h*_f_*/h* rises especially beyond 0.25. To avoid material overuse and ensure cost-effective design, it is recommended that the side-bonding height not exceed 0.25 times the beam depth. This recommendation aligns with the provision in GB 50367-2013, which restricts side bonding to the bottom quarter of the beam’s depth within the tension zone.
(2) This paper presents a calculation method for the flexural capacity of reinforced concrete beams strengthened with carbon fiber-reinforced polymer sheets bonded to their side surfaces using different configurations. Furthermore, two approaches for determining the reduction factor *η*_f_ in the flexural capacity calculation of CFRP-strengthened beams with bottom-surface bonding are proposed: one based on the provisions of the relevant design code, and the other derived from a newly developed quadratic trend function.By comparing the results of three calculation methods with experimental values, it was found that the errors of all three calculation methods were very small; especially when the ratio of the pasting height to the beam height (*h*_f_/*h*) exceeds 0.5,it is recommended that the amplification factor *η*_f_ be determined using the proposed quadratic trend function for calculating the flexural capacity of RC beams strengthened with CFRP sheets bonded to the bottom surface.
(3) In flexural capacity calculations for side-bonded CFRP-strengthened beams, the strength utilization factor *ψ*_f_ is introduced to account for the fact that actual tensile strain in the CFRP sheet often falls short of the design value. Conventionally, *ψ*_f_ is capped at 1.0 when exceeding this threshold. However, both the code-based and quadratic *η*_f_ based soffit-bonding calculation methods herein relax this constraint allowing *ψ*_f_ > 1.0 to offset losses associated with effective area reduction caused by non-uniform strain development.
(4) The ultimate flexural capacity of CFRP side-bonded reinforced concrete beams increases with either the side-bonding ratio hf/h or the CFRP sheet thickness *t*; however, beyond a certain critical value of *h*_f_/*h* or *t*, the ultimate flexural capacity begins to decrease,a phenomenon attributed to premature debonding or inefficient stress transfer.
